# Circulating Angiogenic T Cells and Their Subpopulations in Patients with Systemic Lupus Erythematosus

**DOI:** 10.1155/2016/2842143

**Published:** 2016-03-15

**Authors:** Jinlin Miao, Feng Qiu, Tingting Li, Peng Zhao, Kui Zhang, Minghua Lv, Jun Wan, Xiaokun Qi, Ping Zhu

**Affiliations:** ^1^Department of Clinical Immunology, Xijing Hospital, Fourth Military Medical University, Xi'an 710032, China; ^2^Department of Neurology, Chinese Navy General Hospital, Beijing 100048, China; ^3^Department of Geriatric Gastroenterology, Chinese People's Liberation Army General Hospital, Beijing 100853, China; ^4^Department of Hematology and Rheumatology, Hanzhong 3201 Hospital, Medicine School of Xi'an Jiaotong University, Hanzhong 723000, China

## Abstract

*Objective*. Systemic lupus erythematosus (SLE) is associated with accelerated atherosclerosis and increased cardiovascular risk. Angiogenic T cells (Tang), a specific T cell subset, have been identified and involved in the repair of damaged endothelium. This study aimed to analyze the Tang cell subsets in relation to disease specific features from SLE patients.* Methods*. Tang cell subsets were assessed in peripheral blood samples from 41 SLE patients and 22 healthy controls (HC) by flow cytometry on the basis of CD31 and CXCR4 expression on CD3+, CD4+, and CD8+ T cells.* Results*. The percentage of circulating CD8+CD31+CXCR4+ T cells (CD8+ Tang), but not CD3+CD31+CXCR4+ T cells (Tang) and CD4+CD31+CXCR4+ T cells (CD4+ Tang), in SLE was higher than HC. The percentages of Tang cell subsets in anti-dsDNA-positive SLE patients were significantly increased as compared to their negative counterparts and HC. Additionally, the levels of circulating Tang cell subsets were negatively correlated with age at sampling and at diagnosis, but not disease duration or disease activity.* Conclusion*. Anti-dsDNA-positivity may identify a group of SLE patients with increased Tang cell subsets and circulating CD8+ Tang cells may be viewed as a potentially useful biomarker of endothelial damage and cardiovascular risk in SLE.

## 1. Introduction

Systemic lupus erythematosus (SLE) is a common autoimmune disease characterized by a dysregulation of the immune system that leads to chronic systemic inflammation [1, 2]. As previous reported, SLE is associated with accelerated atherosclerosis and increased risk of cardiovascular events driving high morbidity and mortality rates [3, 4]. However, the endothelial damage, accelerated atherosclerosis, and increased cardiovascular disease observed in SLE patients cannot be fully explained by traditional cardiovascular risk factors or the use of corticosteroids [5]. It has been proposed that systemic inflammation, dysregulated cytokine profile, and altered T cell subsets play important roles in endothelial dysfunction and increased cardiovascular risk presenting in SLE patients [6]. Therefore, identification of cardiovascular damage and repair biomarkers related to SLE may reveal insights into pathogenesis and may be used to monitor the cardiovascular risk and improve the cardiovascular health.

Recently, a specific T cell population, termed angiogenic T cells (Tang), has been described that promotes the formation of new blood vessels and repair of damaged endothelium by cooperating with endothelial progenitor cells (EPCs) [7]. EPCs are a heterogeneous population participated in vasculogenesis and vascular repair [8]. Furthermore, Hur and colleagues showed that Tang cells are characterized by the expression of CD3, the platelet endothelial cell adhesion molecule (CD31), and the receptor for stromal-derived factor 1 (CXCR4) [7]. As a subset of CD3+ T cells, Tang cells also express CD4 or CD8. It has been reported that decreased Tang cell frequencies are associated with vascular disease [9], and circulating Tang cells are decreased in patients with RA, an autoimmune disease that has increased prevalence of cardiovascular events [10]. However, little was known about the percentages of Tang cell subsets (including CD3+CD31+CXCR4+, CD4+CD31+CXCR4+, and CD8+CD31+CXCR4+ cells) in circulation of SLE patients.

Therefore, we explore whether the percentages of Tang cell subsets measured by flow cytometry are different in patients with SLE comparing with healthy controls (HC). Moreover, we aim to assess their potential link to clinical and laboratory abnormalities.

## 2. Materials and Methods

### 2.1. Patients and Controls

Samples of peripheral blood (PB) were obtained from 41 SLE patients and 22 HC. All patients fulfilled the American College of Rheumatology Revised Criteria for Classification of SLE [11]. Disease activity was assessed by systemic lupus erythematosus disease activity index (SLEDAI) score at the time of recruitment. None of the patients had a history of coronary artery disease, diabetes, or cardiac insufficiency. Other information of patients' demographic, clinical, and immunological manifestations and current medications is described in Table 1. Informed consent was obtained from all subjects. This study was approved by the Ethics Committee of Xijing Hospital and conforms to the recommendations of the Declaration of Helsinki.

### 2.2. Flow Cytometric Analysis

PB mononuclear cells (PBMCs) were isolated from sodium heparinized whole blood by Ficoll-Paque density gradient centrifugation (GE Healthcare, Pittsburgh, PA, USA). Then, the phenotypes of lymphocytes were determined using flow cytometry. Briefly, PBMCs were stained with the following fluorochrome conjugated monoclonal antibodies: anti-CD3-peridin chlorophyll protein (Percp), anti-CD4-Percp, anti-CD8-Percp, anti-CD31-phycoerythrin (PE), anti-CXCR4-allophycocyanin (APC), and isotype-matched control IgG antibodies (all from BD Biosciences, San Diego, CA, USA) for 30 min at room temperature, according to the manufacturer's instructions. After being washed with PBS, a minimum of 20,000 events per tube was acquired using a FACSCalibur flow cytometer (BD Biosciences) and analyzed using CellQuest software (BD Biosciences) and FlowJo 7.6.1 software (Tree Star).

### 2.3. Statistical Analysis

Differences between groups were determined using the nonparametric Mann-Whitney *U* test. Correlations were evaluated by nonparametric Spearman's rank correlation analysis. For all tests, a two-sided *p* value less than 0.05 was considered significant. Data analyses were performed using SPSS 17.0 software (SPSS, Chicago, IL, USA).

## 3. Results

### 3.1. Baseline Characteristics of Subjects

Clinical and laboratory characteristics of SLE patients and HC are illustrated in Table 1. A total of 42 patients with SLE and 22 HC were recruited into the study. There was no significant difference in gender and age between SLE patients and HC. The traditional cardiovascular risk factors have been reported to have an inverse relationship with the circulating Tang cells [7]. In this study, the group of SLE patients and HC did not differ significantly in cardiovascular risk factors, including smoking, diabetes, hyperlipidemia, and hypertension (all *p* > 0.05).

### 3.2. Percentage of Circulating Tang Cell Subsets in SLE Patients and HC

**Figure 1:**
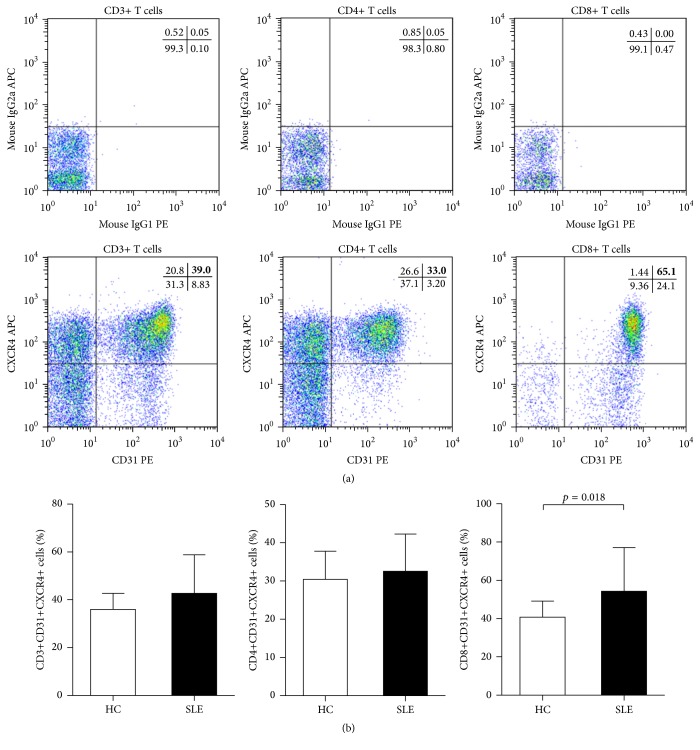
FACS analysis of circulating Tang cell subsets. (a) Typical examples of CD31 and CXCR4 expression after gating on CD3+, CD4+, and CD8+ T cells from peripheral blood of representative patients with SLE. Quadrants were set according to the fluorescence signal provided by the isotype-matched control IgG antibodies. (b) Percentages of circulating Tang cell subsets (including CD31+CXCR4+ cells in CD3+, CD4+, and CD8+ T cells) in SLE patients and HC are shown. *p* values were assessed by Mann-Whitney* U* test.

As shown in Figure 1(a), representative examples of CD31 and CXCR4 expression in CD3+, CD4+, and CD8+ T cells were analyzed by flow cytometry. The percentages of circulating CD31+CXCR4+ cells in total CD3+ T cells (Tang) and CD31+CXCR4+ cells in CD4+ T cells (CD4+ Tang) tended to be slightly higher in SLE patients when compared to HC; however, the differences did not reach statistical significance (Figure 1(b)). What is more, the percentages of CD31+CXCR4+ cells in CD8+ T cells (CD8+ Tang) were significantly increased in patients with SLE as compared to HC (Figure 1(b)).

### 3.3. Autoantibody Status Was Associated with the Levels of Tang Cell Subsets

**Figure 2:**
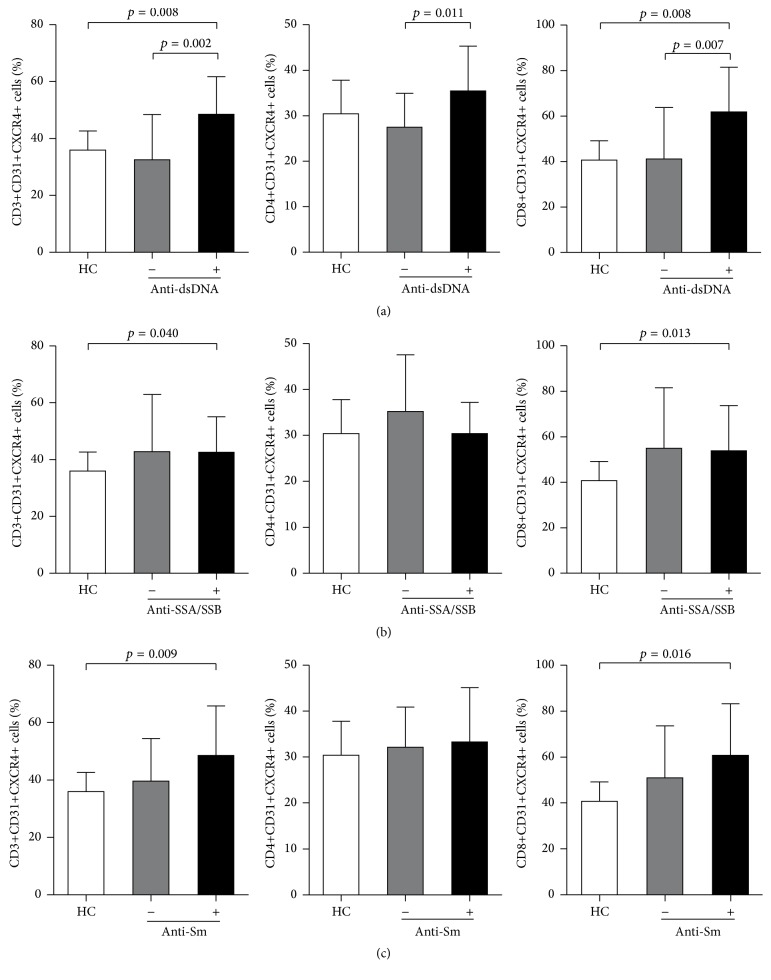
Association between Tang cell subsets and autoantibody status. Percentages of circulating Tang cell subsets (including CD31+CXCR4+ cells in CD3+, CD4+, and CD8+ T cells) in SLE patients with or without anti-dsDNA autoantibodies (a), anti-SSA/SSB autoantibodies (b), and anti-Sm autoantibodies (c). *p* values were assessed by Mann-Whitney* U* test.

Then, we wanted to determine whether alterations in the percentages of circulating Tang cell subsets were associated with autoantibody status in SLE patients. The most striking observation came after subdividing SLE patients into two groups based on the anti-dsDNA antibody status. The percentage of Tang, CD4+ Tang, and CD8+ Tang in anti-dsDNA-positive SLE patients was significantly increased when compared with their negative counterparts and HC (Figure 2(a)). And the percentage of Tang and CD8+ Tang in anti-dsDNA-positive, anti-SSA/SSB-positive, or anti-Sm-positive SLE patients was significantly increased as compared to HC. However, no differences in circulating Tang cell subsets were observed between anti-SSA/SSB-positive or anti-Sm-positive SLE patients and their negative counterparts (Figures 2(b) and 2(c)).

### 3.4. Correlations of Tang Cell Subsets with Clinical Features in SLE Patients

The correlation of circulating Tang cell subsets with clinical features in SLE patients was shown in Table 2. In order to assess the relationship between Tang cell subsets and disease activity in SLE, we performed a correlation between the percentages of Tang, CD4+ Tang, CD8+ Tang, and SLEDAI scores. No significant correlation was found between Tang cell subsets and SLEDAI scores (Table 2). Additionally, the percentages of circulating Tang, CD4+ Tang, and CD8+ Tang were negatively correlated with age at sampling and age at diagnosis, but not disease duration (Table 2).

## 4. Discussion

Growing evidences indicate that angiogenesis and angiogenic factors critically contribute to the pathogenesis of SLE [12–14]. Indeed, in vitro and in vivo experiments showed that Tang cells were required for colony formation and differentiation of early EPCs and were supposed to stimulate the endothelial cells by secreting angiogenic cytokines [7]. Furthermore, Tang cells enhanced endothelial cell proliferation and function, suggesting that Tang cells may be viewed as a biomarker for cardiovascular risk [7]. Therefore, in this study, the percentages of circulating Tang cell subsets, including Tang, CD4+ Tang, and CD8+ Tang, were comprehensively assessed by flow cytometry in SLE patients.

Similar to a recent study [15], our data showed that the percentage of circulating Tang cells in SLE patients was not different from HC. However, about the circulating Tang cell levels, discrepant results have been reported. Lopez et al. [10, 15] showed that the percentage of Tang cells is only about 8% in HC, RA, and SLE patient. In agreement with the data of a recent study [7], we showed that the circulating Tang level was about 30–40% in HC and SLE patients. In fact, the population studied in our and Kim et al. work [7] comprised Asians, whereas the studies of Lopez et al. [10, 15] recruited Europeans. Therefore, discrepancies in Tang levels may be due to the various ethnic groups. We believe that further investigations with a larger number of patients and with follow-up observation are needed to confirm and extend the current results.

Meanwhile, we found that not only CD3+ cells, but also CD4+ and CD8+ T cells express CD31 and CXCR4 on their surface. Thus, the levels of circulating CD4+ Tang and CD8+ Tang cells were also examined in this study. There were no significant differences in the percentage of CD4+ Tang cells between SLE patients and HC. However, the percentage of circulating CD8+ Tang cells was significantly increased in SLE patients when compared to HC, suggesting enhanced CD8+ Tang cells may be a repair response to the endothelial damage present in SLE patients. In addition, a defective functionality has been reported about Tang cells from SLE patients, including cytotoxic phenotype and accelerated senescence [15]. Consequently, a deficient vascular repair might further enhance CD8+ Tang cell levels.

As previously reported, several autoantibodies, including anti-dsDNA, anti-SSA/SSB, and anti-Sm antibodies, play important roles in the diagnosis, disease activity evaluation, organ involvement, and prognosis evaluation of SLE [16]. Moreover, it has been reported that autoantibody status is associated with vasculopathy and cardiovascular risk in SLE [17] and other diseases [18–20]. In the present work, among SLE patients with positive antibodies, both the Tang and CD8+ Tang cell percentages were significantly increased as compared to HC. Interestingly, after dividing these patients according to the presence or absence of antibodies, anti-dsDNA-positive SLE patients, but not anti-SSA/SSB-positive or anti-Sm-positive patients, demonstrated increased levels of Tang, CD4+ Tang, and CD8+ Tang cells as compared to their negative counterparts and HC. Therefore, these data indicate that anti-dsDNA-positivity may isolate a subset of SLE patients associated with endothelial damage and at higher risk of vasculopathy.

We did not find any statistically significant correlations between Tang cell subsets and disease duration or SLE activity according to SLEDAI. As reported in the literatures [7, 10, 15], Tang cell levels were correlated inversely with age in HC, RA, and SLE patients. In line with these results, we showed that Tang and their subpopulations CD4+ Tang and CD8+ Tang cell levels were negatively correlated with age at sampling and age at diagnosis. These findings supported that late age is associated with defective endothelial repair and cardiovascular risk, and Tang cell subsets may be used as a biological marker for cardiovascular disease [7].

## 5. Conclusion

Our results demonstrated that the level of CD8+ Tang is elevated in SLE patients comparing with HC. And the presence of autoantibody, especially for anti-dsDNA antibodies, may identify a group of SLE patients with increased Tang cell subsets and higher risk of vasculopathy. These findings suggest that, for the first time, circulating CD8+ Tang cells may be viewed as a potentially useful biomarker of endothelial damage in SLE. Further studies are required to clarify the function of CD8+ Tang cells and investigate the mechanism for their change in SLE.

## Figures and Tables

**Table 1:** Clinical and laboratory characteristics of SLE patients and healthy controls (HC).

Characteristics	HC	SLE
Number of patients	22	41
Female sex, *n* (%)	20 (90.9)	38 (92.7)
Age at sampling, median (IQR), years	31.5 (25.0–34.3)	30.0 (24.0–42.0)
Age at diagnosis, median (IQR), years	NA	25.0 (20.0–38.7)
Disease duration, median (IQR), years	NA	4.0 (1.0–9.0)
Clinical features		
SLEDAI, median (IQR)	NA	12.0 (8.0–14.0)
ESR, mm/hour, median (IQR)	NA	29.0 (12.0–45.0)
CRP, mg/dL, median (IQR)	NA	1.1 (0.3–2.1)
Positivity of anti-dsDNA, *n* (%)	NA	26 (63.4)
Positivity of anti-Sm, *n* (%)	NA	23 (56.1)
Positivity of anti-SSA/SSB, *n* (%)	NA	14 (34.1)
Skin symptoms, *n* (%)	NA	21 (51.2)
Neurological symptoms, *n* (%)	NA	2 (4.9)
Haematological symptoms, *n* (%)	NA	23 (56.1)
Renal disorder, *n* (%)	NA	9 (22.0)
Arthritis, *n* (%)	NA	28 (68.3)
Cardiovascular risk factors, *n* (%)		
Smoking	2 (9.1)	3 (7.3)
Diabetes mellitus	0 (0)	0 (0)
Hyperlipidemia	3 (13.6)	7 (17.1)
Hypertension	4 (18.2)	9 (22.0)
Treatment, *n* (%)		
None or NSAIDs	NA	5 (12.2)
Antimalarial drugs	NA	32 (78.0)
Glucocorticoids	NA	16 (39.0)
Immunosuppressive drugs^a^	NA	6 (14.6)

Values are presented as median (interquartile range, IQR) or number (percentage). SLEDAI, SLE disease activity index; ESR, erythrocyte sedimentation rate; CRP, C-reactive protein; NSAIDs, nonsteroidal anti-inflammatory drugs; NA, not applicable; ^a^azathioprine, mycophenolate mofetil.

**Table 2:** Correlations between the percentages of Tang cell subsets and SLEDAI, disease duration, age at sampling, or age at diagnosis in SLE patients.

	Tang (%)		CD4+ Tang (%)		CD8+ Tang (%)	
	*r*	*p*	*r*	*p*	*r*	*p*
SLEDAI	−0.215	0.177	−0.260	0.101	−0.151	0.345
Disease duration	0.112	0.487	−0.120	0.454	0.042	0.795
Age at sampling	−0.455	**0.003**	−0.471	**0.002**	−0.461	**0.002**
Age at diagnosis	−0.444	**0.004**	−0.349	**0.025**	−0.396	**0.010**
